# Application of a high-throughput microcrystal delivery system to serial femtosecond crystallography

**DOI:** 10.1107/S1600576720002423

**Published:** 2020-03-25

**Authors:** Donghyeon Lee, Sehan Park, Keondo Lee, Jangwoo Kim, Gisu Park, Ki Hyun Nam, Sangwon Baek, Wan Kyun Chung, Jong-Lam Lee, Yunje Cho, Jaehyun Park

**Affiliations:** a Department of Mechanical Engineering, POSTECH, 77 Cheongam-Ro, Pohang, 37673, Republic of Korea; b PAL-XFEL, Pohang Accelerator Laboratory, 80 Jigok-ro 127 beongil, Pohang, 37673, Republic of Korea; c College of Life Sciences and Biotechnology, Korea University, 145 Anam-ro, Seoul, 02841, Republic of Korea; d Institute of Life Science and Natural Resources, Korea University, 145 Anam-ro, Seoul, 02841, Republic of Korea; e Department of Materials Science and Engineering, POSTECH, 77 Cheongam-Ro, Pohang, 37673, Republic of Korea; f Department of Life Sciences, POSTECH, 77 Cheongam-Ro, Pohang, 37673, Republic of Korea

**Keywords:** high-throughput microcrystal delivery, HT-MCD, microcrystal delivery systems, serial femtosecond crystallography, X-ray free-electron lasers, XFELs

## Abstract

Microcrystal delivery methods are pivotal in the use of serial femtosecond crystallography (SFX) to resolve the macromolecular structures of proteins. Here, the development of a novel technique and instruments for efficiently delivering microcrystals for SFX are presented. The new method, which relies on a one-dimensional fixed-target system that includes a microcrystal container, consumes an extremely low amount of sample compared with conventional two-dimensional fixed-target techniques at ambient temperature. This novel system can deliver soluble microcrystals without highly viscous carrier media and, moreover, can be used as a microcrystal growth device for SFX. Diffraction data collection utilizing this advanced technique along with a real-time visual servo scan system has been successfully demonstrated for the structure determination of proteinase K microcrystals at 1.85 Å resolution.

## Abstract 2

A new one-dimensional fixed-target sample delivery method combined with a real-time visual servo scan system has been developed for serial femtosecond crystallography. The developed high-throughput microcrystal delivery system shows significant improvements for microcrystal delivery efficiency at an optimal crystal stability and significantly reduced sample consumption to reveal protein structures utilizing X-ray free-electron laser pulses.

## 1. Introduction   

X-ray free-electron laser (XFEL) facilities have enabled the collection of diffraction data for macromolecular structural study with minimal radiation damage at ambient temperature (Barends *et al.*, 2014 ▸; Neutze *et al.*, 2000 ▸; Boutet *et al.*, 2012 ▸; Chapman *et al.*, 2011 ▸; Liu *et al.*, 2013 ▸). Serial femtosecond crystallography (SFX) is one of the emergent techniques used to determine the ångström-resolution structures of macromolecules using XFEL sources (Spence *et al.*, 2012 ▸). An essential prerequisite for SFX is a sample delivery instrument to position fresh micro- or nanosized crystals at the interaction point with the XFEL pulses.

Several sample delivery systems have been developed for that purpose, such as liquid jet injectors using the gas dynamic virtual nozzle (DePonte *et al.*, 2008 ▸, 2011 ▸), electrospinning (Sierra *et al.*, 2012 ▸), lipidic cubic phase (LCP) injectors (Weierstall *et al.*, 2014 ▸), acoustic injectors (Roessler *et al.*, 2016 ▸), viscous media (Kovácsová *et al.*, 2017 ▸; Sugahara *et al.*, 2015 ▸; Park *et al.*, 2019 ▸; Conrad *et al.*, 2015 ▸) and goniometer-based scanning (Cohen *et al.*, 2014 ▸). A liquid jet injector typically delivers soluble crystals contained in a buffer solution at a flow rate of 10–30 µl min^−1^ (Deponte *et al.*, 2011 ▸, 2008 ▸). This technique produces a low background signal but has a high sample consumption of up to 10–100 mg of the protein at LCLS (Weierstall *et al.*, 2014 ▸). The LCP injector significantly reduces the sample consumption to approximately 100 µl (∼0.5 mg) to obtain the full data set (Weierstall *et al.*, 2014 ▸). However, a bubble-free sample with an appropriate viscosity is required for stable sample injection. The compatibility of LCP with the precipitant solution of crystallization must be checked in advance for data collection with the LCP injector (Fromme *et al.*, 2015 ▸). Physically, the delicate microcrystals can be fractured during the mechanical mixing process in the syringes. In addition to free-standing dynamic liquid delivery methods, fixed sample scanning techniques are also applied for carrying out SFX experiments (Sherrell *et al.*, 2015 ▸; Oghbaey *et al.*, 2016 ▸; Roedig *et al.*, 2017 ▸); for example, microcrystals have been subjected to two-dimensional spreading into silicon chips or between two sandwiched films (Doak *et al.*, 2018 ▸; Hunter *et al.*, 2014 ▸). Such methods provide a simple sample preparation scheme; however, they also require additional efforts to achieve highly viscous environments or the mechanical attachment of thin films to prevent dehydration. Thus, it would greatly facilitate SFX data collection to develop a highly efficient crystal delivery system that overcomes the limitations described above. We report the development of a novel one-dimensional fixed target system composed of a microcrystal container (MCC) and delivery instruments that facilitate high-throughput SFX experiments. The high-throughput microcrystal delivery (HT-MCD) system presented here combines the advantages of previous sample delivery methods such as the liquid jet, LCP injection and two-dimensional fixed-target methods by ensuring soluble crystal stability in an optimal buffer, significantly reducing sample consumption and providing a convenient preparation protocol. Moreover, the system does not require laborious preliminary sample optimization processes before data collection and can be used as a microcrystal growth device itself.

## 2. High-throughput microcrystal delivery system   

**Figure 1:**
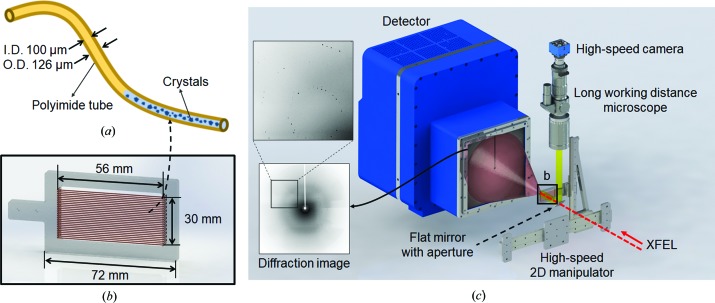
(*a*) Microcrystal container (ID: 100 µm; OD: 126 µm). Blue-coloured protein crystal solution is packed in the polyimide tube. (*b*) MCC chip design. A single MCC chip can contain 27 lines of the polyimide tube [shown in (*a*)]. Four MCC tubes can be installed at the same time. (*c*) Schematic of experimental setup. The real-time visual servo system consists of a high-speed camera, long working distance microscope and high-speed 2D manipulators. The MCC chip position is precisely manipulated on the basis of the fast image process. The inset shows one of the diffraction images acquired with a Rayonix MX225-HS detector.

Protein microcrystals are contained in an MCC made of micro polyimide tubing (length: 500 mm; Furukawa Electric Co. Ltd) [Fig. 1 ▸(*a*)], which provides highly stable conditions with the appropriate buffer. The inner diameter of the MCC is 100 µm and the thickness is 13 µm. This inner diameter allows the accommodation of incoming crystal sizes below 40 × 40 × 40 µm and clusters of smaller crystals. It even permits the crystals to remain in the growth condition when the crystal solution is injected. Therefore, during a sample delivery operation, the MCC does not cause the clogging problems that can be encountered with dynamic sample delivery techniques such as the gas dynamic virtual nozzle liquid jet injector or LCP injector (Weierstall *et al.*, 2014 ▸). Instead, this method combines the advantages of both delivery methods with optimal hydration and a controllable sample consumption rate. The cylindrical shape of this system generates random orientation of the microcrystals more effectively than two-dimensional multi-window holders, which have geometrical limitations such as window size and roughness (Hunter *et al.*, 2014 ▸; Cohen *et al.*, 2014 ▸; Zarrine-Afsar *et al.*, 2012 ▸). Nevertheless, large non-spherical crystals can settle into preferred orientations relative to the wall of the tube in the MCC system.

Protein microcrystals can be mounted in the MCC using a mixed crystal solution injection system, which is composed of polyetheretherketone (PEEK) tubing and fittings consisting of unions, ferrules and nuts. At one end, polyimide tubing is inserted into the PEEK tubing (OD: 1/16 in ≃ 1.59 mm; ID: 150 µm; IDEX Health & Science) and fixed with a fast-drying adhesive. The above part is connected through a union to an RN-type Hamilton 1710 series gas-tight syringe (100 µl) filled with crystal solution using a Hamilton standard needle and sleeve. The MCC chip [Fig. 1 ▸(*b*)] has sufficient hole pairs to hold four microtubes. The crystal solution volume that can be contained in a single tube is approximately 3.93 µl, and thus a total of approximately 15.7 µl of crystal solution can be used for a chip with four tubes. Data collection with one MCC chip is fully sufficient to resolve a protein structure (Supporting Table S1).

### 2.1. MCC chip and crystal mounting   

The MCC chip is made with an acrylic plate and can contain four polyimide tubes simultaneously. The length of each array is 56 mm; thus, XFEL shots can be directed at 1080 spots in an array with 50 µm spatial intervals [Fig. 1 ▸(*a*)]. One end of each polyimide tube is inserted into a PEEK tube and fixed with a quick-drying glue (Loctite 401 adhesive). After the connection is firmly fixed, the tube structure is connected to a union fitting with a PEEK ferrule.

The Hamilton syringe and the tubing are connected with a standard Hamilton needle and sleeve and a 10–32 coned nut (F-331; IDEX Health & Science). A schematic drawing of the connection scheme is presented in Supporting Fig. S1. The crystal solution is injected into the tubing by pushing the plunger. When the crystal solution injection is finished, both ends of the polyimide tubing are closed with the glue. A photograph of the MCC chip containing four polyimide tubes is shown in Supporting Fig. S2.

### 2.2. Real-time visual servo method for MCC chips   

Owing to the tiny size of the MCC tubing, the XFEL interaction point is strongly affected by the mechanical alignment during the scan. When the tubing or chip is misaligned by an angle as small as 0.1°, if the initial beam position is centred in the lateral direction, the XFEL beam will be aimed outside the tubing by 52 µm after only 30 mm of travel. Therefore, diffraction data cannot be collected from the other half of the tubing region without additional corrections. Consequently, real-time position correction is important for full data collection and minimization of the missing region. For fast position correction, we have embedded a visual servo system for position monitoring and control. The visual servo system uses real-time optical images to obtain the position information of the tubing array. The extracted position information is fed back to the fast stage controller for accurate delivery of the tubing array in real time. A schematic drawing of the scan system is shown in Fig. 1 ▸(*c*).

**Figure 2:**
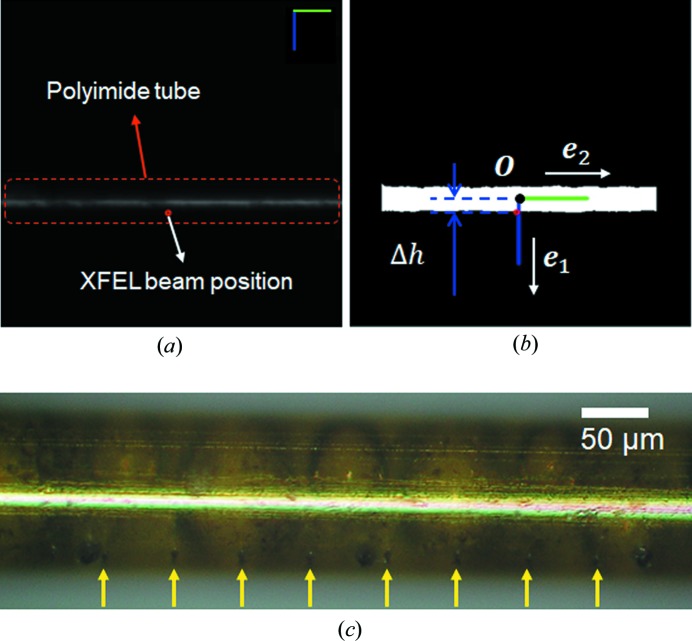
Micro polyimide tube tracking with real-time visual feedback. (*a*) Captured image of the MCC using the vision acquisition module. The scale bars are shown in the upper right corner (the longitudinal bar indicates 100 µm length and the axis direction of the piezo actuator). (*b*) Processed image with binary conversion. The controller with a fast feedback signal helps to maintain the distance between *O* and the XFEL beam position, Δ*h*. (*c*) Photograph of the MCC after completion of the experiment. The focused XFEL pulses generate tiny holes and gas bubbles via radiation damage. The distance between adjacent holes, indicated by yellow arrows, is 50 µm.

The operation of the real-time position feedback program is based on the processing of images acquired with a long working distance microscope (working distance: 300 mm) [Fig. 2 ▸(*a*)]. The details of the chamber setup are presented in Supporting Fig. S3. The acquired image is converted into a binary image by using simple image processing algorithms [Fig. 2 ▸(*b*)]. A Gaussian filter is applied to the image to alleviate speckle point noise, and the image is then converted into a binary image with a constant threshold value. As a result, the polyimide tube is detected as a long rectangular blob in the binary image. From the detected blob, the centroid position [*O* in Fig. 2 ▸(*b*)] is calculated by averaging the position of each blob pixel. The coordinates along the lateral and longitudinal axes of the blob [*e*_1_ and *e*_2_ in Fig. 2 ▸(*b*)] are calculated by applying principal component analysis (PCA). The Gaussian filter, image thresholding and PCA algorithm are implemented using a well known open-source library, *OpenCV* (Bradski, 2000 ▸). Then, the calculated values are transformed into the actuator coordinates to make the array follow a desired trajectory while correcting the lateral offset value.

#### 2.2.1. Fast visual servo scan system   

Sample delivery using the MCC chip is performed within the HT-MCD sample chamber on the basis of information from the visual servo scan system, which consists of several modules: a high-speed position manipulation system, a fast vision acquisition system and other miscellaneous parts. The detailed structure of the sample chamber is presented in the supporting information (supporting information text S1, S2 and Supporting Figs. S3, S4 and S5).

The XFEL illumination point on the MCC chip is accurately controlled with a high-speed position-manipulation stage, which consists of piezo-based actuators (*X* axis: SmarAct SLLV42, 470 mm travel; *Y* axis: SmarAct SLL12, 270 mm travel) for rapid and exact two-dimensional motion. The maximum speed and position accuracy are 20 mm s^−1^ and 50 nm. The full speed allows raster scanning for XFEL pulse repetition up to 400 Hz with a 50 µm illumination separation. A flat mirror (TopRaySys, Republic of Korea) with an aperture (size: 3 mm) at the centre is installed with a 45° tilt angle to acquire sample images by a vision acquisition system (Supporting Fig. S4).

The fast vision acquisition system captures real-time images around the XFEL beam illumination position by using an ultra-long working distance microscope (UWZ-300, working distance: 300 mm; UNION Optical Co. Ltd, Japan) and a high-speed CMOS camera (mvBlueCOUGAR-XD, Matrix Vision, Germany).

## 3. Experiments and results   

### 3.1. Sample preparation and diffraction data acquisition   

Microcrystals of proteinase K were prepared according to procedures similar to those used in previous work (Masuda *et al.*, 2017 ▸). Proteinase K from *Tritirachium album* was purchased from Sigma (P2308). The microcrystals were produced by mixing 50 µl of the protein solution (80 mg ml^−1^ protein concentration in 20 m*M* MES pH 6.5) and 50 µl of the crystallization solution [0.25 *M* sodium nitrate, 0.05 *M* calcium chloride and 0.1 *M* MES (pH 6.5)] at 291 K. After 30 min, the crystal solution was gently mixed by pipetting to accelerate the nucleation of the microcrystals. In one or two hours, micro-sized crystals (15 × 15 × 15 µm on average) were successfully obtained. The microcrystals were transferred directly to the MCC before the SFX experiment.

We performed the SFX experiments at the NCI PAL-XFEL experimental station (Park *et al.*, 2016 ▸; Kang *et al.*, 2017 ▸; Ko *et al.*, 2017 ▸). The X-ray wavelength was 1.28 Å (9.7 keV), very near the maximum photon flux energy region. The XFEL pulse duration and energy were approximately 20 fs and 500 µJ, corresponding to approximately 1–2 × 10^11^ photons per pulse at 9.7 keV. The bandwidth of the incident energy spectrum for the pink beam is approximately 0.2%. The size of the focused beam obtained by the Kirkpatrick–Baez mirrors was approximately 4 × 8 µm at the sample position, and the focal distance was approximately 5.68 m from the centre of the two mirrors (Kim *et al.*, 2018 ▸). The diffraction data were monitored by *OnDA* (Mariani *et al.*, 2016 ▸) and collected with an MX225-HS detector (Rayonix, LLC, Evanston, IL, USA) (supporting movie). The 4 × 4 binning mode was utilized to match the XFEL repetition rate of 30 Hz. The pixel size was 156 × 156 µm for that binning mode. The distance between the sample and detector was 133 mm.

Even though a chip has 27 hole pairs, to conveniently attach fittings to the tubes, 24 lines are usually installed for the crystal delivery with the chip. Proteinase K microcrystals (15 × 15 × 15 µm on average) in buffer solution were injected into the MCCs using a Hamilton syringe (100 µl). The crystal number density used here was adjusted to 5 × 10^7^ crystals ml^−1^ (Supporting Fig. S6). The chip position was remotely controlled with a stage controller located near the chamber that can manipulate the interaction intervals for each XFEL pulse and sample consumption rate by adjusting the moving speed of the stage. The scan speed was set to obtain XFEL beam illumination separation of 50 µm to avoid damaging effects of adjacent XFEL shots (Lee *et al.*, 2019 ▸). Every XFEL pulse made a tiny hole in the tubing and generated gas bubbles via radiation damage [Fig. 2 ▸(*c*)] (Lee *et al.*, 2019 ▸; Meents *et al.*, 2010 ▸). The diffraction peaks from proteinase K crystals were clearly recorded at low background scattering except for weak circular diffraction signals from polyimide (Supporting Fig. S7). In total, we collected 366 604 images to obtain the proteinase K structure, as shown in Table 1 ▸. The hit rate was 39% (143 539 images), and 73 138 images were successfully indexed (Table 1 ▸). In particular, we noticed that even the data collected from a single chip are sufficient to determine the protein structure. For a single MCC chip, approximately 65 000 images were collected, and the number of hit images was approximately 29 000 (39% hit rate). Of all hit images, 14 680 images (51%) were successfully indexed (Supporting Table 1). The data collection statics from one MCC chip in the HT-MCD system indicate that the CC*, *R*_split_, signal-to-noise (S/N) ratio and completeness are sufficient for determining a protein structure even in the case of a low quantity of the crystal sample. By contrast, when we performed the LCP sample delivery method (LCP beam diameter: 100 µm) (Park *et al.*, 2018 ▸), 40 µl of monoolein-mixed proteinase K crystal solution (5 × 10^7^ per ml) were insufficient to determine the protein structure and refine the model, as shown in Supporting Table 1.

### 3.2. Structure determination, refinement and analysis   

The collected diffraction data were filtered with *Cheetah* (version 8; Barty *et al.*, 2014 ▸) and processed with the program *CrystFEL* (version 0.6.3; White *et al.*, 2012 ▸ 2016 ▸). The parameters for peak detection were optimized for *Cheetah*. The experimental geometry was also refined for *CrystFEL*. The parameter min-snr used for peak detection during peak finding was 4. Indexing was performed using *DirAx* (version 1.17; White *et al.*, 2012 ▸) with peak integration parameters of int-radius = 3, 4, 5. The measured diffraction intensities were merged with process_hkl in the *CrystFEL* suite (White *et al.*, 2012 ▸). The structure of *T. album* proteinase K was determined via the molecular replacement method using *PHENIX* (version 1.14–3260) (Adams *et al.*, 2010 ▸) by employing a model of *T. album* proteinase K as a search model (PDB code 4b5l; J. Jakoncic, V. Stojanoff & V. Honkimaki, unpublished). To avoid model bias during the molecular replacement method, we used the search model without water and ligand molecules. Water molecules and the ligands (Ca^2+^ ions) were added using *Coot* (version 0.8.9; Emsley *et al.*, 2010 ▸) and manually inspected on the basis of both 2*mFo*–*DFc* and *mFo*–*DFc* maps. Water molecules and calcium ions were placed in positions corresponding to the density map, where positive peaks were higher than 1.8σ and 4.0σ in the *mFo*–*DFc* map, respectively. The MR model was first refined with a rigid-body protocol and simulated annealing using *phenix.refine*. After a few cycles of restrained refinement, further refinement of the model was performed with *PHENIX* (Liebschner *et al.*, 2019 ▸), and the model was evaluated using *MolProbity* (version 4.4; Chen *et al.*, 2010 ▸). The crystals of *T. album* proteinase K belonged to the tetragonal space group *P*4_3_2_1_2, with unit-cell parameters of *a* = *b* = 68.54 Å, *c* = 108.38 Å, α = β = γ = 90°. Table 1 ▸ summarizes the data collection statistics.

### 3.3. Comparative analysis among proteinase K structures   

We determined the crystal structure of *T. album* proteinase K using the HT-MCD technique. The structure was refined against 30–1.85 Å data to *R*_work_ and *R*_free_ values of 0.196 and 0.239. Diffraction is geometrically limited by the chamber window size. Therefore, we could collect data for a resolution of up to 1.85 Å. The structure accounts for 279 amino acid residues in one monomer, two Ca^2+^ ions and 207 water molecules in the asymmetric unit.

**Figure 3:**
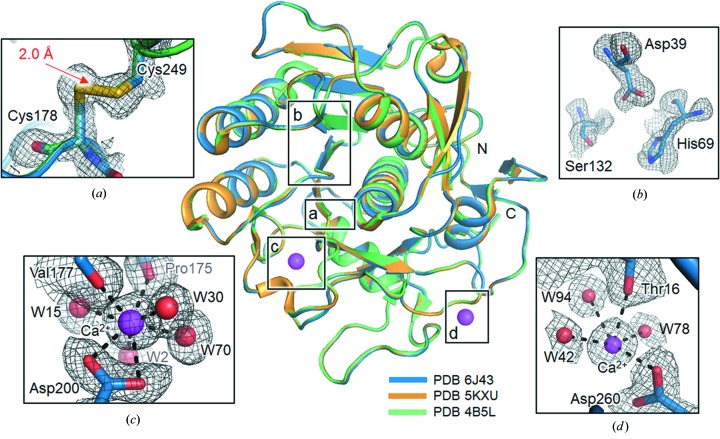
Overall structure of *T. album* proteinase K. Three proteinase K structures (PDB code 6j43 in blue; PDB code 5kxu in orange; PDB code 4b5l in green) are presented as ribbon diagrams. In panels (*a*), (*b*), (*c*) and (*d*), detailed structures are drawn with *mFo*–*DFc* electron density maps, which are contoured at 2.0 σ (in grey mashes). The disulfide (Cys178 and Cys249) is indicated as a red arrow in panel (*a*). In panel (*b*), the catalytic triad (Ser–His–Asp) is depicted at its active site. Two Ca^2+^ ions with interacting residues and water molecules are shown in panels (*c*) and (*d*). Ca^2+^ ions and water molecules are coloured in magenta and red, respectively. The structures were constructed using *PyMOL* (DeLano, 2002 ▸).

Proteinase K (EC 3.4.21.64) is a subtilisin-like serine protease possessing a catalytic triad (Ser–His–Asp) at its active site and an oxyanion hole (Betzel *et al.*, 1988 ▸; Masuda *et al.*, 2017 ▸). Proteinase K is an enzymatic catalyst of hydrolysis and aminolysis (Betzel *et al.*, 1988 ▸; Masuda *et al.*, 2017 ▸). Proteinase K has two Ca^2+^-binding sites, as shown in Fig. 3 ▸ (Betzel *et al.*, 1988 ▸).

We compared our determined structure (PDB code 6j43) with previous ones obtained using XFEL (PDB code 5kxu; Masuda *et al.*, 2017 ▸) and synchrotron sources (PDB code 4b5l) at room temperature and found that our result is essentially identical to those from both 5kxu (an r.m.s. deviation of 0.11 Å for 279 equivalent Cα positions) and 4b5l (an r.m.s. deviation of 0.13 Å for 279 equivalent Cα positions) (Fig. 3 ▸).

### 3.4. Ca^2+^-binding sites, disulfide bonds and active sites among the three proteinase K structures   

Of the two Ca^2+^-binding sites, the site that coordinated with Pro175 and Asp200 is known to tightly bind the Ca^2+^ ion. The Ca^2+^ ion also interacts with four water molecules (W2, W15, W30 and W70) in a pentagonal bipyramidal coordination [Fig. 3 ▸(*c*)]. The other Ca^2+^-binding site is liganded to Thr16, Asp260 and three water molecules (W42, W78 and W94) [Fig. 3 ▸(*d*)]. It is known that Ca^2+^ ions maintain the local structural stability of the substrate-recognition region (Ser132–Gly136), which is regarded as an important factor for the activity of proteinase K (Betzel *et al.*, 1988 ▸). As shown in Figs. 3 ▸(*a*) and 3 ▸(*b*), the geometry of the active site (Asp39, His69 and Ser132) and the disulfide bond between Cys178 and Cys249 are basically similar for the three proteinase K structures. Overall, no substantial differences between the three proteinase K structures (PDB codes 6j43, 5kxu and 4b5l) were observed in the Ca^2+^-binding sites, disulfide bonds or active sites, implying that all three proteinase K structures are stable and active. Indeed, we observed and monitored the activity of proteinase K shown in Supporting Fig. S8. An enzymatic activity assay of proteinase K was performed using the protease activity assay kit (AB112153, Abcam). For the assay, the total assay volume was 100 µl, and 24 n*M* proteinase K (P2308, Sigma) was used. The enzymatic reaction was performed at 295 K and monitored with a Tecan Infinite fluorimeter F200 (Ex/Em = 540/590 nm).

## 4. Discussion   

We have developed a highly efficient microcrystal delivery system and determined the structure of proteinase K using this system. Our new sample delivery method optimally hydrates and stably preserves the sample; therefore, several MCC chips can be prepared in advance. The prepared chip can be loaded very easily and quickly (in approximately 2 min) when the chip exchanging port of the current chamber is open, which can save time in the preparation of the target sample. No additional matrix carrier or crystal distribution procedures on the membrane are required. The statistics for the collected data from one to four MCC chips indicate that one can potentially determine a protein structure even with the data collected from a single MCC chip (Table 2 ▸ and Supporting Table 1). The overall CC* values are over 0.95 and the *R*_split_ values are between 24.10 and 11.25% for one to four MCC chips. The overall signal-to-noise ratio is 3.96, even in the case of a single MCC chip. For the crystals that are hardest to produce, such as membrane protein crystals, the HT-MCD system could be an alternative way to solve the protein structure.

**Figure 4:**
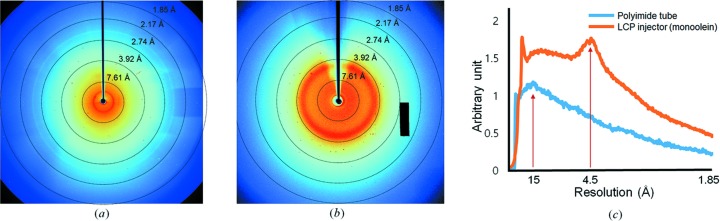
Comparison of background scattering between the MCC and LCP injector containing proteinase K crystals. (*a*) This image presents the X-ray diffraction quality of the tiny proteinase K crystals (5 × 5 × 5 µm crystals). The resolution reaches approximately 4 Å. The polyimide tube shows diffuse X-ray scattering at approximately 15 Å. (*b*) The LCP system shows high X-ray scattering at approximately 4.0 Å. A small area outside of the assembled chips in the Rayonix MX225-HS shows a hardware issue, *i.e.* the analogue-to-digital unit (ADU) values are saturated (shown by the rectangular black box). (*c*) Comparison of background scattering when X-rays hit the polyimide tube and monoolein-containing sample. As shown in panels (*a*) and (*b*), the scattering noise from our method is strongly reduced compared with that from the LCP injector.

The tubing is filled with microcrystal suspension fluid and sealed to prevent buffer evaporation or leakage. Additionally, the inner diameter of the tubing is too small to allow a fluid flow inside. Under such a static configuration, gravitational force and the diffusive effect that occurs because of Brownian motion can be applied to the suspended crystals. The spatial stability of crystals can be approximately predicted from the ratio of gravitational to Brownian forces as 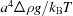 (Larson, 1999 ▸; Tanner, 2000 ▸). Here, *a* is the spherical particle radius, 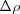 is the density difference between the dispersed and continuous phases, *g* is the acceleration due to gravity, 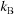 is the Boltzmann constant, and *T* is the temperature. If this ratio is higher than 1, the particles can settle. Here, the density of the proteinase K crystal is estimated as follows. The volume per unit cell of the proteinase K crystal is calculated to be 509 140.15 Å^3^ (5.09 × 10^−19^ cm^3^). The mass of one unit cell, which is composed of six monomers (40 300 Da per monomer), is approximately 4.02  × 10^−19^ g. When we add water (2.00 × 10^−19^ g) to the protein, the total mass becomes 6.02 × 10^−19^ g. Therefore, the crystal density is calculated to be approximately 1.18 g cm^−3^. When the particle is 14 µm in diameter with a density difference of 0.18 at room temperature (298 K), the ratio value is approximately 1.03. This result implies that, for crystals larger than 14 µm, the effect of gravity is dominant, and the crystals can settle at the bottom of the tubing if other external forces are negligible. In this case, data collection will be more efficient if the XFEL beam position is aligned below the vertical centre of the tubing. Here, we aligned the XFEL pulse interaction point at approximately 30 µm below the centre of the tubing to focus on large crystals settled near the bottom of the tube. At 45 µm from the centre of the tubing, the diffraction limit decreased from 1.85 to 2.5 Å without significant background scattering (supporting information text S3 and Supporting Fig. S9). In addition, we compared the background scattering level from the MCC setup with that from another sample delivery technique using smaller proteinase K crystals (∼5 × 5 × 5 µm crystals). The scattering for the MCC setup showed a relatively low intensity [Fig. 4 ▸(*a*)] compared with that for the delivery medium (monoolein) in an LCP injector [Fig. 4 ▸(*b*)] at similar flow diameter (∼100 µm). Here, the X-ray interaction points were set to the centres of the tubing and the LCP sample flow, respectively. Furthermore, an MCC setup containing similar-sized lysozyme crystals in an environment with a high salt concentration (3.5 *M* NaCl) showed a clear diffraction pattern at a reasonably low background noise level (Supporting Fig. S10).

**Figure 5:**
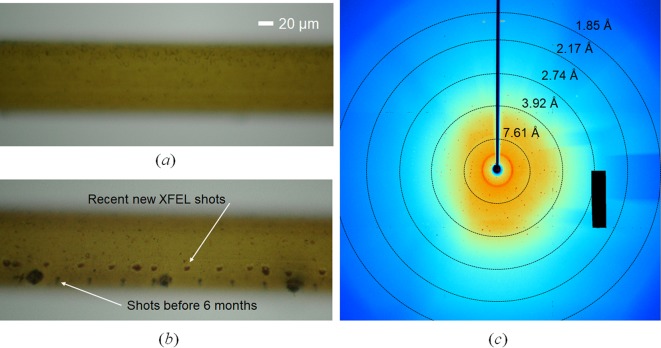
Previously used proteinase K crystals in tubes for SFX experiments can be preserved without severe dehydration or damage, and the MCC tube can be used as a microcrystal growth device. (*a*) Proteinase K crystals were produced in the tube after direct injection of both proteinase K and crystallization solution. These findings suggest that the HT-MCC tube can be used as a container for microcrystal growth. (*b*) Two kinds of XFEL shots can be seen in this figure (recent shots and 6 month shots). (*c*) Diffraction image from proteinase K crystals preserved in a previously used tube.

One of the important advantages of our method is that the tube can be used as a microcrystal growth device, in which crystals are produced in the tube after the direct injection of a crystallization solution [Fig. 5 ▸(*a*)]. In the case of proteinase K, we observed a high density of microcrystals (10–20 µm) in the tube after a day when we injected proteinase K with a macrocrystal-growing crystallization solution directly into the tube. Moreover, we found that the previously used tubes for SFX experiments preserved the proteinase K crystals without dehydration or severe damage after 6 months. As shown in Fig. 5 ▸, we could collect diffraction images from the previously used tubes despite previous XFEL shots [Fig. 5 ▸(*b*) and (*c*)]. Therefore, researchers can use this distinctive feature of the MCC when they need to preserve small quantities of important and sensitive protein crystals.

As another application, we believe that the HT-MCD technique can also be applied to serial millisecond crystallography (SMX) at a synchrotron (Martin-Garcia *et al.*, 2017 ▸; Stellato *et al.*, 2014 ▸; Weinert *et al.*, 2017 ▸) with an adequate scanning system that is suited to the beamline conditions. A previous study using the SMX technique showed successful data collection. In that study, a long exposure time (85 ms on the GM/CA 23-ID-D beamline at the Advanced Photon Source, USA) was required to achieve a resolution of 2.0 Å for lysozyme crystals of 5–10 µm (Martin-Garcia *et al.*, 2017 ▸). Moreover, the average crystal travel speeds corresponding to exposure times ranged from 120 to 1550 µm s^−1^ (Martin-Garcia *et al.*, 2017 ▸). If the scanning speed is appropriately controlled near the ranges described above, this technique can be another option for delivering protein crystals without cryocooling conditions. On the other hand, we have observed that the MCC is stable enough to obtain diffraction patterns at ∼100 K, thus showing the potential usefulness of the MCC in diffraction experiments at temperatures down to that of liquid nitrogen (Supporting Fig. S11). In addition, the MCC can serve as a handling medium for microcrystals with somewhat viscous media; for example, a gel-like sample consisting of a 1:1 mixture of proteinase K crystals and monoolein could be inserted into the MCC. Since the phase of monoolein is quite sensitive to humidity (Ganem-Quintanar *et al.*, 2000 ▸), this developed technique can make sample handling easier and help to avoid damage caused by relatively low humidity environments.

In conclusion, the HT-MCD method makes significant contributions to overcoming the bottlenecks of microcrystal delivery for SFX, namely, crystal dissolution and fractures caused by the matrix carrier, high sample consumption and background noise signals, in serial crystallography experiments. Moreover, we envision that the MCC could be an easily accessible device for handling microcrystals with minimal effort and will significantly increase the efficiency of determining protein structures utilizing SFX at XFEL facilities.

## Supplementary Material

Supporting information file. DOI: 10.1107/S1600576720002423/yr5056sup1.mp4

Supporting information file. DOI: 10.1107/S1600576720002423/yr5056sup2.pdf

PDB reference: *Tritirachium album* proteinase K 6j43

## Figures and Tables

**Table 1:** Statistics for data collection, phasing and model refinement statistics for proteinase K Values in parentheses refer to the highest-resolution shell. The CC* value is described by Karplus & Diederichs (2012 ▸); CC* = [2CC_1/2/_(1 + CC_1/2_)]^1/2^.

Data collection			
Space group		*P*4_3_2_1_2	
Unit-cell length (Å)		*a* = 68.54, *b* = 68.54, *c* = 108.38	
Unit-cell angle (°)		α = 90.0, β = 90.0, γ = 90.0	
X-ray wavelength (Å)		1.2782	
No. of collected images		366 604	
No. of hits		143 539	
No. of indexed images		73 138	
Indexing rate from hits (%)		51.0	
No. of merged images		73,138	
Resolution range (Å)		30.0–1.85 (1.88–1.85)	
Total/unique reflections		26 806 279/42 177	
Redundancy		635.6 (266.1)	
Completeness (%)		100.0 (100.0)	
CC*		0.994 (0.966)	
〈*I*/*σ_I_*〉		9.4 (3.5)	
*R*_split_ (%)		9.7 (26.1)	

Model refinement			
Resolution range (Å)		30.0–1.85 (1.88–1.85)	
*R*_work_/*R*_free_ (%)		19.6/23.9	
Wilson *B* factor		21.4	
No. of non-H atoms/average *B* factor (Å^2^)			
Protein		2052/5.78	
Water		207/21.9	
Ca^2+^ ion		2/9.34	
R.m.s. deviations from ideal geometry			
Bond lengths (Å)/bond angles (°)		0.006/0.764	
PDB code		6j43	
Ramachandran plot (%)			
Favoured/outliers		95.7/0.0	
Rotamer outliers		1.4	

**Table 2:** Statistics for the collected data from one to four MCC chips Values in parentheses refer to the highest-resolution shell. The statistics as a function of resolution range are given in Supporting Table 1.

No. of chips used	CC*	*R*_split_ (%)	S/N ratio	Completeness (%)
1	0.9647 (0.8368)	24.10 (60.18)	3.959 (1.60)	100 (100)
2	0.9839 (0.9449)	15.87 (33.48)	5.850 (2.76)	100 (100)
3	0.9886 (0.9601)	13.17 (27.56)	7.081 (3.36)	100 (100)
4	0.9918 (0.9721)	11.25 (23.44)	8.239 (3.89)	100 (100)
