# Supplementary material for: Application of a high-throughput microcrystal delivery system to serial femtosecond crystallography
Source: J Appl Crystallogr. 2020 Mar 25;53(Pt 2):477–85. doi: 10.1107/S1600576720002423 (PMC7133064; doi:10.1107/S1600576720002423)
Supplement: Supplementary file 2 [file j-53-00477-sup2.pdf]

JOURNAL OF
APPLIED
CRYSTALLOGRAPHY

Volume 53 (2020)

Supporting information for article:

Application of a high-throughput microcrystal delivery system to serial femtosecond crystallography

Donghyeon Lee, Sehan Park, Keondo Lee, Jangwoo Kim, Gisu Park, Ki Hyun Nam, Sangwon Baek, Wan Kyun Chung, Jong-Lam Lee, Yunje Cho and Jaehyun Park

S1. HT-MCD chamber configuration

The chamber body is manufactured from aluminium plates and is 480 × 320 × 510 mm^3. The chamber contains an L-shaped major plate that supports the high-speed delivery module and a 45° tilted mirror. The mirror position is adjusted by 2D motorized linear stages attached to the major plate (KGB40A-B1-S-50S, travel range: 50 mm; repeatability: 20 µm; ERAEtech, Republic of Korea). An aluminium plate with a 1-mm pinhole aperture is installed at the entrance of the chamber to reduce parasitic noise from upstream around the XFEL beam, and the pinhole plate position is adjusted by the same type of 2D motorized linear stage used for mirror manipulation. To prevent damage to the detector by ultra-strong XFEL pulses, a beam stopper is installed at the back wall of the chamber. Two commercial LED light sources (LEDMM2, Misumi, Japan) are mounted at the front wall of the chamber for the vision acquisition module, and another LED (YL-6011, SMG, China) is mounted to an arm connected to a rotational manipulator (AX-12a, ROBOTIS, Republic of Korea) at the back wall of the chamber for sample coordinate setup and alignment.

S2. Sample delivery module controller structure

To track the specific region of the MCC tube, the controller is designed as a double-loop structure: the inner loop is for position regulation (velocity-level PID controller), and the outer loop is for tube tracking (position-level P controller) (Supporting Fig. S5). The inner-loop controller is a low-level controller that controls the velocity of the piezo actuators to maintain a reference velocity ($\boldsymbol{v}_{\text{ref}}$); it calculates the control input of the piezo actuator with the position feedback from the linear encoder. The outer-loop controller calculates the reference velocity of each actuator with visual feedback. Using the calculated tube position information from the processed image, the reference velocity is defined as

$$\boldsymbol{v}_{\text{ref}} = \boldsymbol{v}_{\text{des}} + K_{\text{p}}(\Delta h - h)\boldsymbol{e}1,$$

where h is the lateral distance between the XFEL beam position and the centroid of the detected tube ($\boldsymbol{O}$ in Fig. 2b), and K_{p} is the control gain. The lateral offset Δh is used to align the XFEL beam with the lower part of the MCC tube so that the XFEL pulses can interact with the crystals settled in the lower part of the tube. In our experiment, K_{p} is set to 100, and Δh is set to -30 µm. The desired velocity ($\boldsymbol{v}_{\text{des}}$) of the MCC chip is 1.5 mm/s for a 50-µm interval at a 30 Hz repetition rate in the longitudinal direction.

S3. Settling effect of the crystals in the tube during data collection

We have calculated the path lengths in the polyimide tube with XFEL beam (~ 8 µm) as shown in the following figure. When the XFEL beam passes through just the bottom of the inner space, the total path lengths are between 40 and 76 µm in the figure. Based on the CXRO X-ray database, the expected X-ray transmission values according to those polyimide thicknesses, are very similar with that at the center region within about 2 %. The background signal from polyimide tube will not give a significant effect on the diffraction signals. The detailed description is shown as following figure.

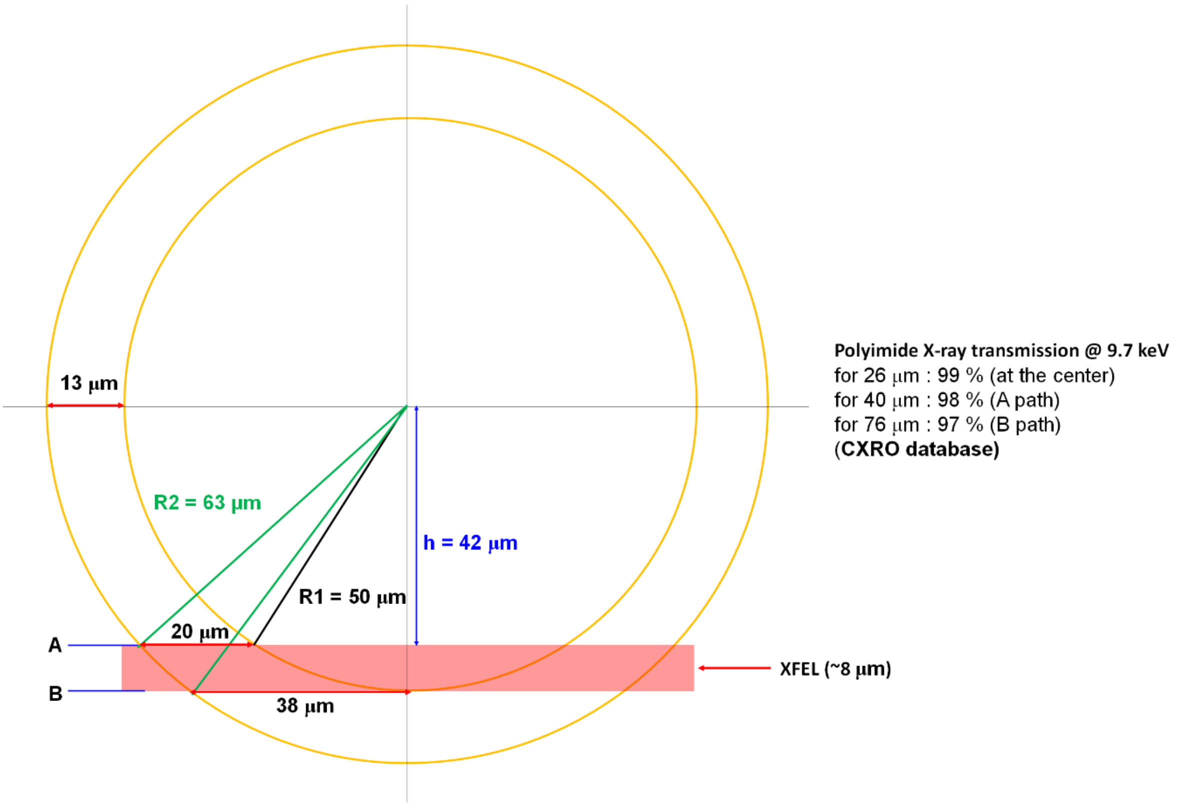

Table S1 Overall statistics of four datasets using the MCC chip as a function of resolution range. Four tables are presented here to show the overall data quality for each data collection.

Statistics of the collected data from one MCC chip

Resolution (Å)	CC*	R_{split} (%)	Signal to noise ratio	Completeness (%)
7.69	0.959392	19.03	5.65	100
3.53	0.954977	18.43	5.71	100
2.95	0.958362	20.18	5.03	100
2.63	0.954589	22.52	4.53	100
2.42	0.954189	24.04	4.19	100
2.26	0.945552	26.72	3.89	100
2.14	0.935414	29.67	3.52	100
2.04	0.926526	34.26	2.98	100
1.95	0.905023	40.36	2.47	100
1.88	0.836802	60.18	1.6	100

Statistics of the collected data from two MCC chips

Resolution (Å)	CC*	R_{split} (%)	Signal to noise ratio	Completeness (%)
7.69	0.981216	13.22	7.91	100
3.53	0.978946	12.53	8.09	100
2.95	0.979223	14.07	7.15	100
2.63	0.979963	14.99	6.54	100
2.42	0.980276	15.87	6.16	100
2.26	0.979247	16.52	5.83	100
2.14	0.973695	18.71	5.33	100
2.04	0.973249	20.36	4.68	100
1.95	0.960342	24.57	4.03	100
1.88	0.94486	33.48	2.76	100

Statistics of the collected data from three MCC chips

Resolution (Å)	CC*	R_{split} (%)	Signal to noise ratio	Completeness (%)
7.69	0.986995	10.83	9.58	100
3.53	0.984515	10.53	9.8	100
2.95	0.985943	11.55	8.66	100
2.63	0.986216	12.33	7.9	100
2.42	0.985541	13.34	7.45	100
2.26	0.985126	13.75	7.05	100
2.14	0.983674	14.97	6.45	100
2.04	0.979154	17.29	5.67	100
1.95	0.97248	20.16	4.88	100
1.88	0.960107	27.56	3.36	100

Statistics of the collected data from four MCC chips

Resolution (Å)	CC*	R_{split} (%)	Signal to noise ratio	Completeness (%)
7.69	0.990338	9.4	11.23	100
3.53	0.989536	8.69	11.47	100
2.95	0.989329	10.04	10.09	100
2.63	0.989452	10.75	9.17	100
2.42	0.99004	11.18	8.65	100
2.26	0.988761	11.92	8.17	100
2.14	0.987409	12.88	7.48	100
2.04	0.985526	14.66	6.56	100
1.95	0.979455	17.23	5.66	100
1.88	0.972126	23.44	3.89	100

Table S2 Statistics of the collected data using the LCP sample delivery method from 40-μl monoolein-mixed proteinase K sample. Number of collected data and hits were 45,494 and 3,365 (hit rates: 7.40%), respectively. Out of total hits, 2,429 images were indexed (indexable rate: 72.2%). Overall CC* and R_{split} were 0.206 and 94.17%, respectively. Crystal density was 5×10^7 per ml.

Resolution (Å)	CC*	R_{split} (%)	Signal to noise ratio	Completeness (%)
7.69	0.4025812	87.43	1.41	100
3.53	0.1434318	88.40	1.36	100
2.95	0.0996396	91.36	1.36	100
2.63	0.2494030	87.76	1.37	100
2.42	0.1912302	92.35	1.33	100
2.26	0.0715391	94.57	1.29	100
2.14	0.2314522	94.92	1.28	100
2.04	0.2315344	96.88	1.24	100
1.95	0.1527985	102.18	1.20	100
1.88	N.D.	107.11	1.16	100

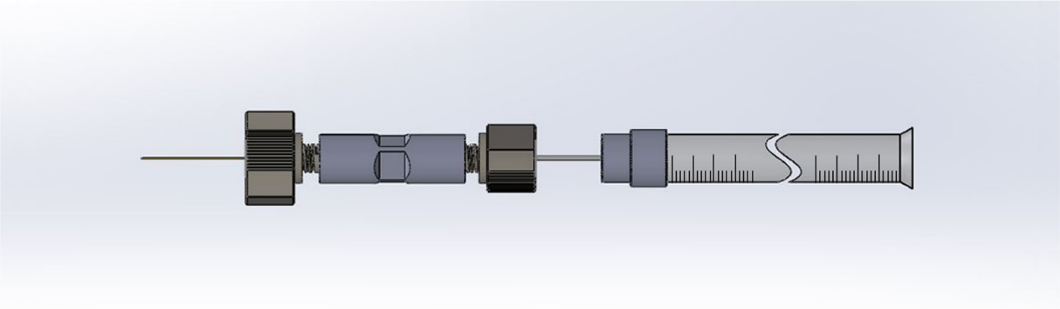

Figure S1 Crystal mounting scheme into the MCC. The injection system is composed of PEEK tubing and fittings consisting of unions, ferrules, and nuts. The dead volume per loading is due to the through-hole volume of the union. The swept volume of the union (P-704, IDEX health and science) is 0.28 μL for a single tube. The total dead volume for one MCC chip corresponds to approximately 1.12 μL for 4 tubes.

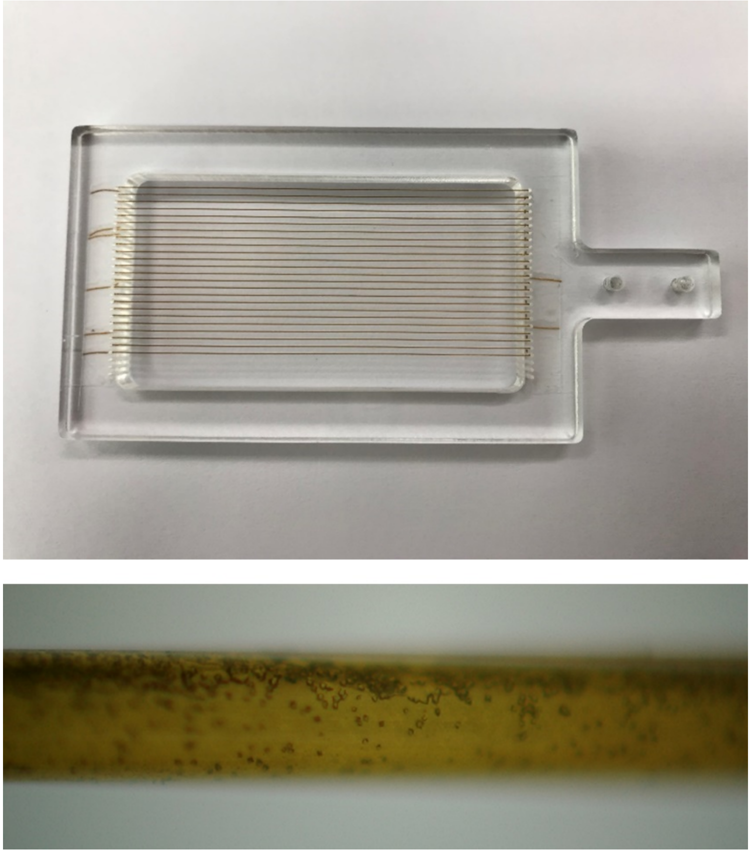

Figure S2 Photo of the MCC composed of four 0.5 m tubes. Each tube makes 6 rows in the chip (upper panel). Microscope image of Proteinase K crystals inside the tube (down panel).

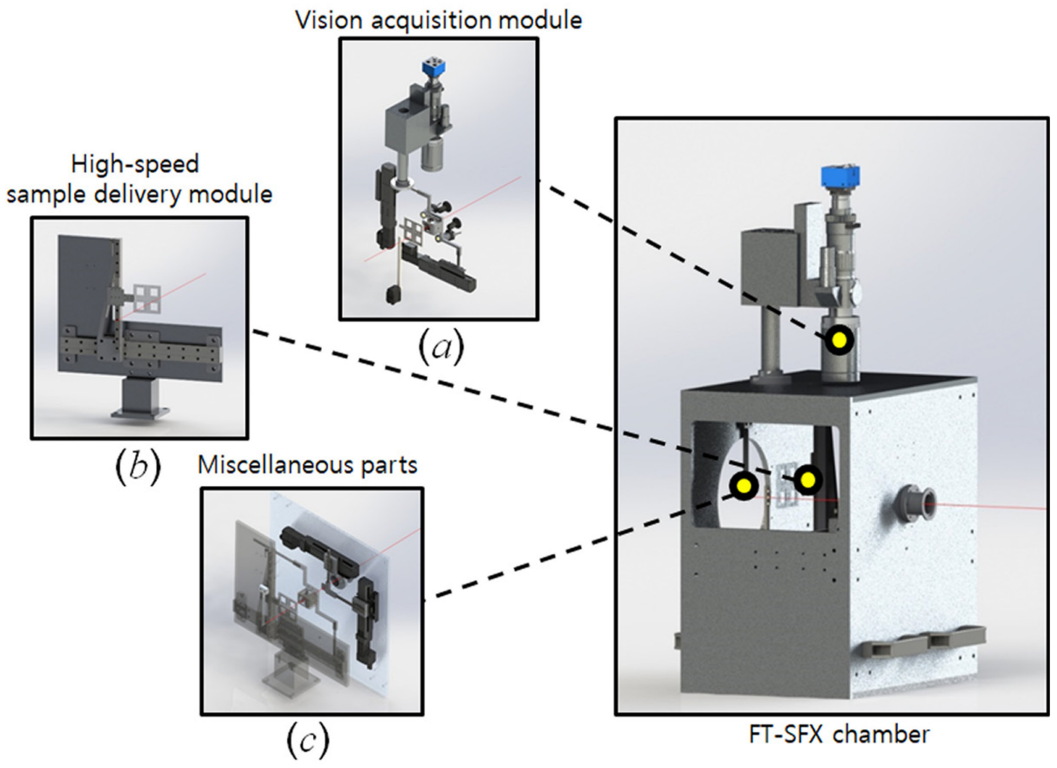

Figure S3 Design of key instruments in the sample chamber for HT-MCD system. (*a*) The vision acquisition module consists of an ultra-long working distance microscope, a high-speed camera, a 45° tilted flat mirror with a circular aperture and LEDs for bright-field imaging. (*b*) The high-speed sample delivery module includes two piezo actuators and a linear guide for MCC chip translation. (*c*) A pinhole plate is installed near the entrance of the chamber to reduce parasitic scattering from upstream, and a beam stopper is installed to block the main XFEL beam and prevent damage to the detector.

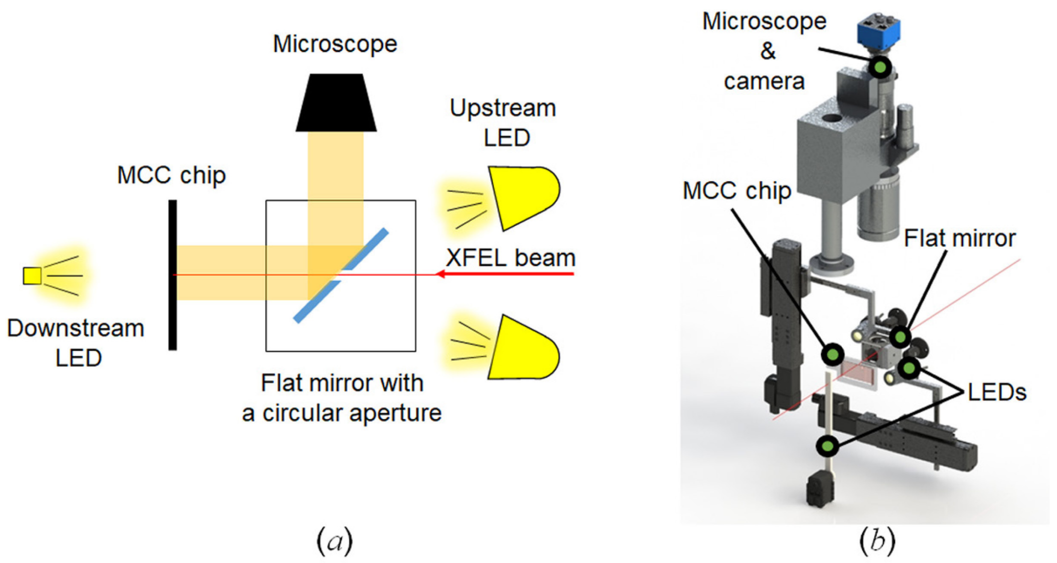

Figure S4 Schematic drawing and close-up view of vision acquisition module. (*a*) Schematic drawing of vision acquisition module. The flat mirror with an aperture at the centre reflects light from the sample array toward the microscopic camera. In the first stage, an LED light is placed behind the sample to retrieve the position coordinates of the MCC chip. During the experiment, the downstream LED is removed, and the upstream LED in front of the mirror is used for chip illumination. (*b*) Close-up view of vision acquisition module. The 2D linear stages manipulate the position of the flat mirror, and a rotational actuator moves the downstream LED in and out of the XFEL beam path.

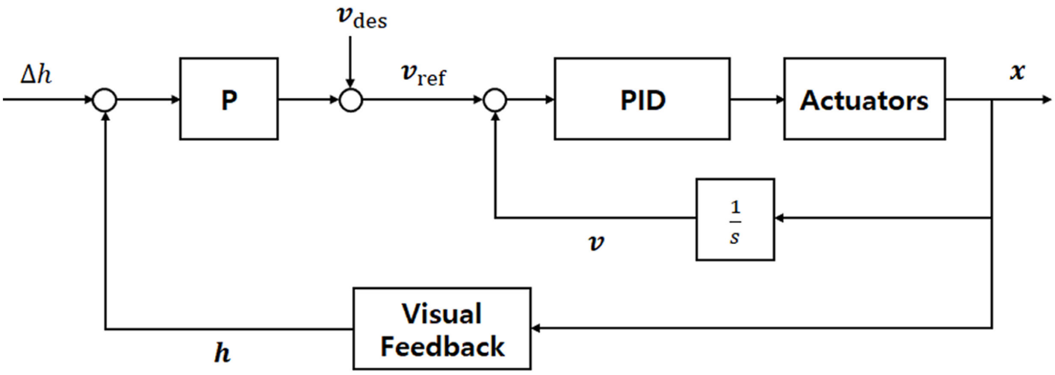

Figure S5 System diagram of the controller for the high-speed sample delivery module. The overall system consists of double-loop control structures. The inner loop PID controller manipulates the velocity of the piezo actuator, and the outer loop regulates the lateral position of the target tube by using the visual feedback. Here, h is the current lateral distance between the detected tube centre and the XFEL position, Δh is the preferred lateral offset between the tube centre and the XFEL beam position, $\boldsymbol{v}_{\mathrm{des}}$ is the desired velocity of the chip, and $\boldsymbol{v}_{\mathrm{ref}}$ is the compensating chip velocity to maintain h at Δh. Here, $\boldsymbol{v}_{\mathrm{ref}}$ is used as a control input of the piezo actuator velocity controller in the inner loop, $\boldsymbol{x}$ is the current 2D position of the piezo actuators, and $\boldsymbol{v}$ is the current velocity of the piezo actuators. From the image captured by the vision acquisition module, an image processing algorithm can extract h when the actuators are located at the target position $\boldsymbol{x}$.

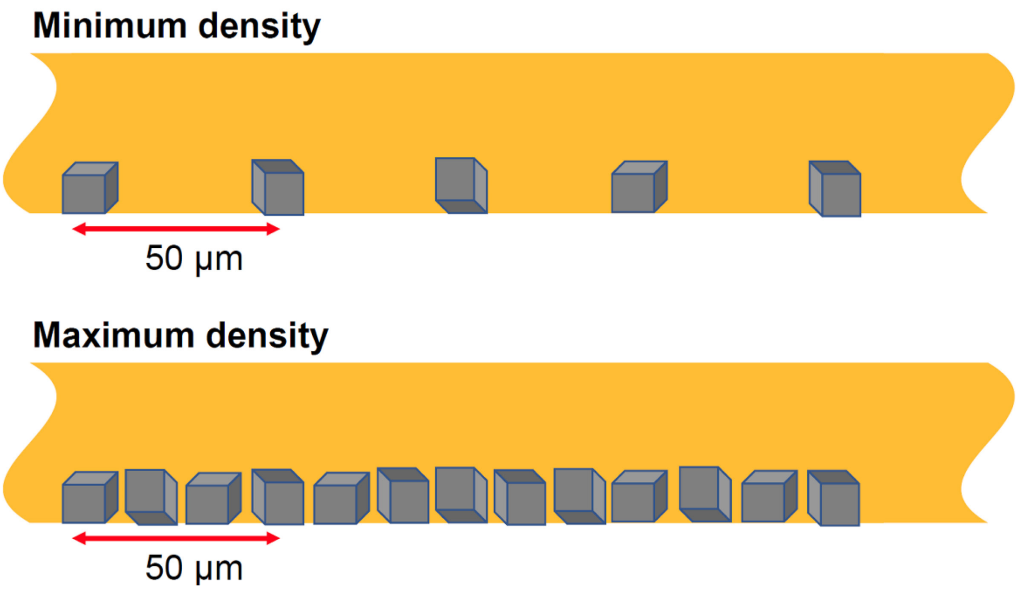

Figure S6 Schematic drawing of ideal crystal densities. The crystal density ranges from 2.5 × 10^6 crystals/ml (minimum density) to 8.4 × 10^6 crystals/ml (maximum density) in the tube with minimized crystal consumption. When the crystals are located at positions that match the scanning step for XFEL pulse illumination, data collection can be performed with high efficiency. We have prepared a sample with a slightly higher crystal density of 5 × 10^7 crystals/ml to achieve more efficient data collection, considering the occurrence of crystal stacking inside the tube.

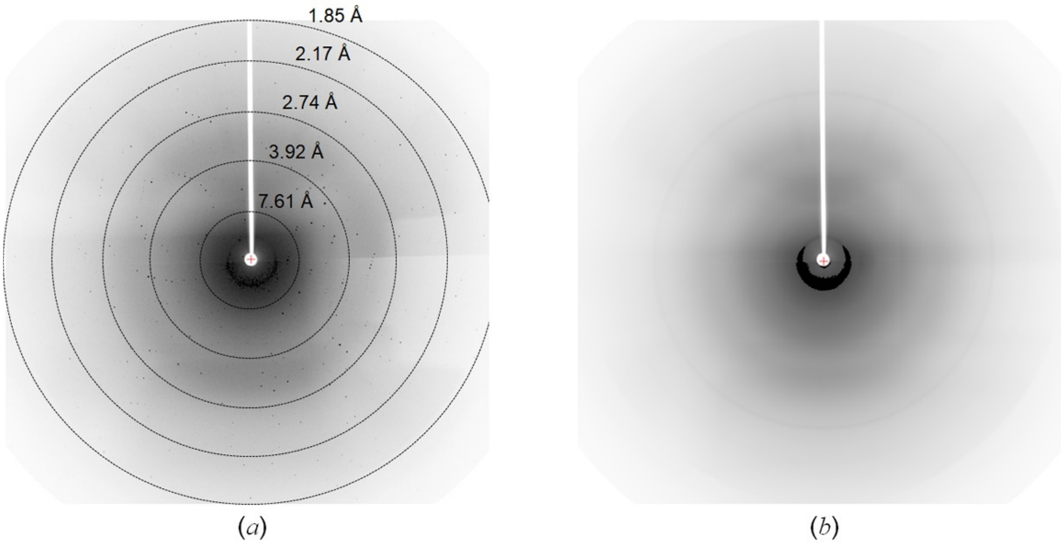

Figure S7 Two X-ray diffraction images showing diffraction resolution and background scattering. (a) X-ray diffraction quality of the proteinase K microcrystals from *T. album*. Resolution rings at 7.61, 3.92, 2.74, 2.17, and 1.85 Å are annotated. (b) X-ray diffraction image acquired without a sample.

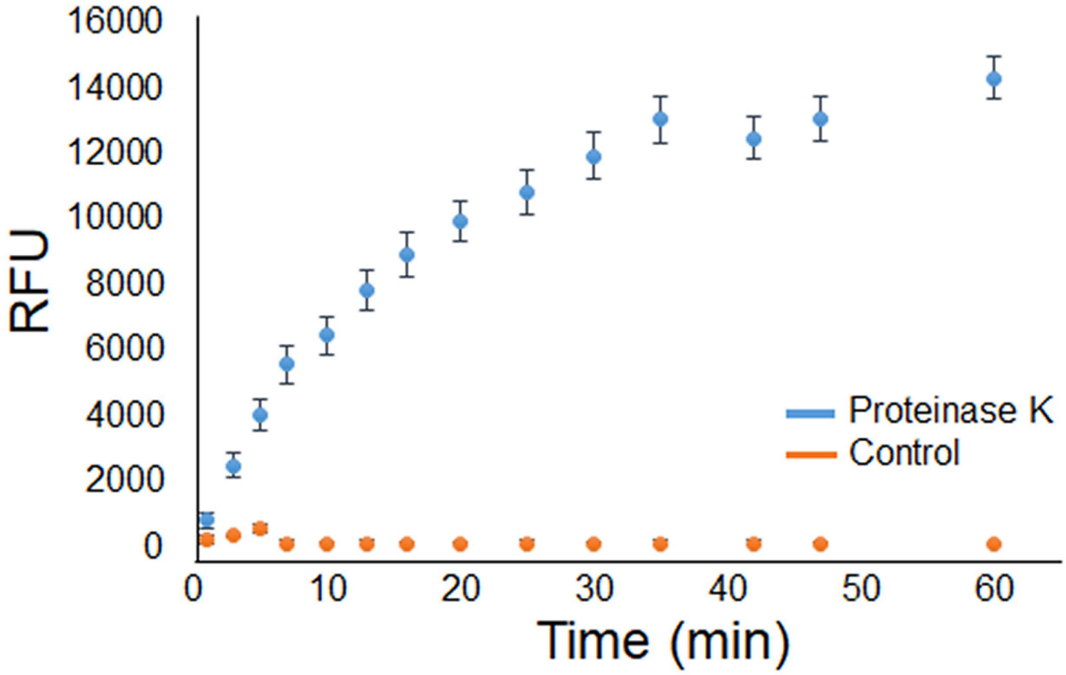

Figure S8 Enzymatic assay result of Proteinase K. The enzyme assay was performed using the protease activity assay kit (AB112153, Abcam). We obtained the activity profile using 24 nM of proteinase K at Ex/Em = 540/590 nm. The enzymatic reaction was performed at 22˚C and monitored by Tecan Infinite fluorimeter F200.

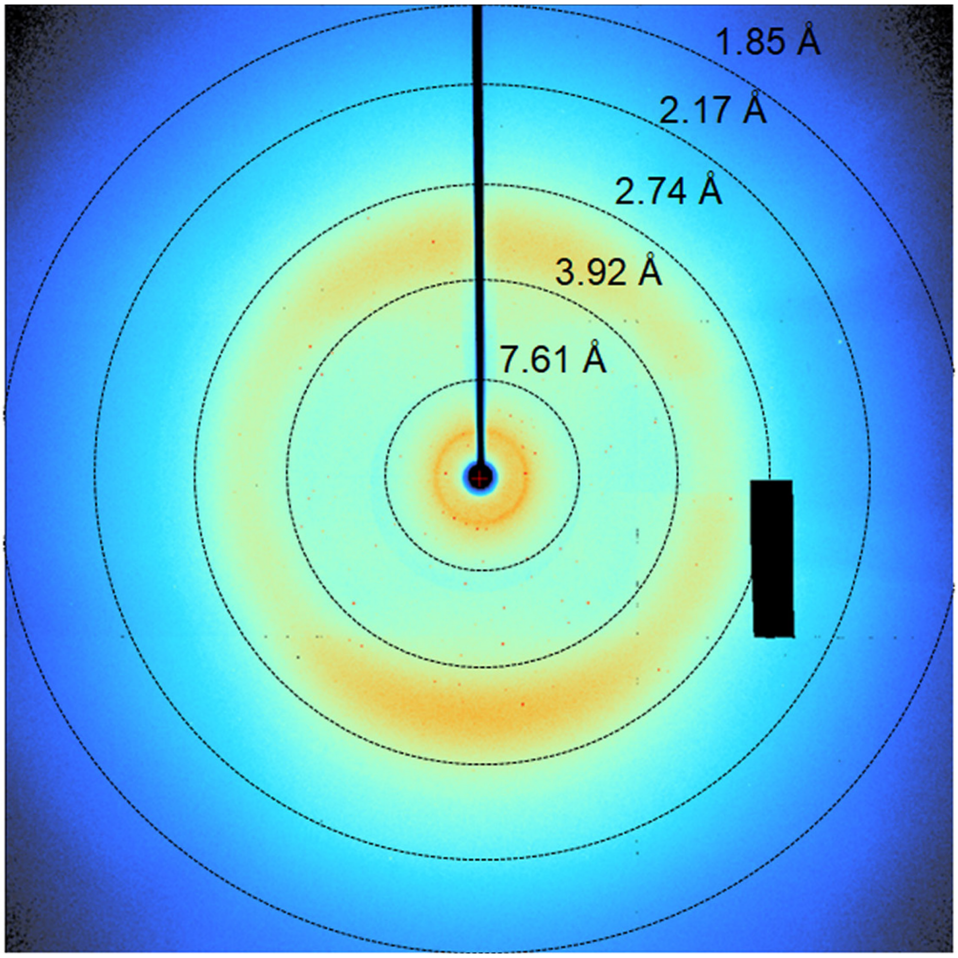

Figure S9 X-ray diffraction quality of the proteinase K microcrystals from *T. album* near the bottom at 45 µm from the center of the tubing. Resolution rings at 7.61, 3.92, 2.74, 2.17, and 1.85 Å are annotated. Although a small area at the right side of the assembled chips in Rayonix MX225-HS has shown a hardware issue, i.e., the ADU values are saturated (shown as the rectangular black box), there is no problem with the collection of SFX data.

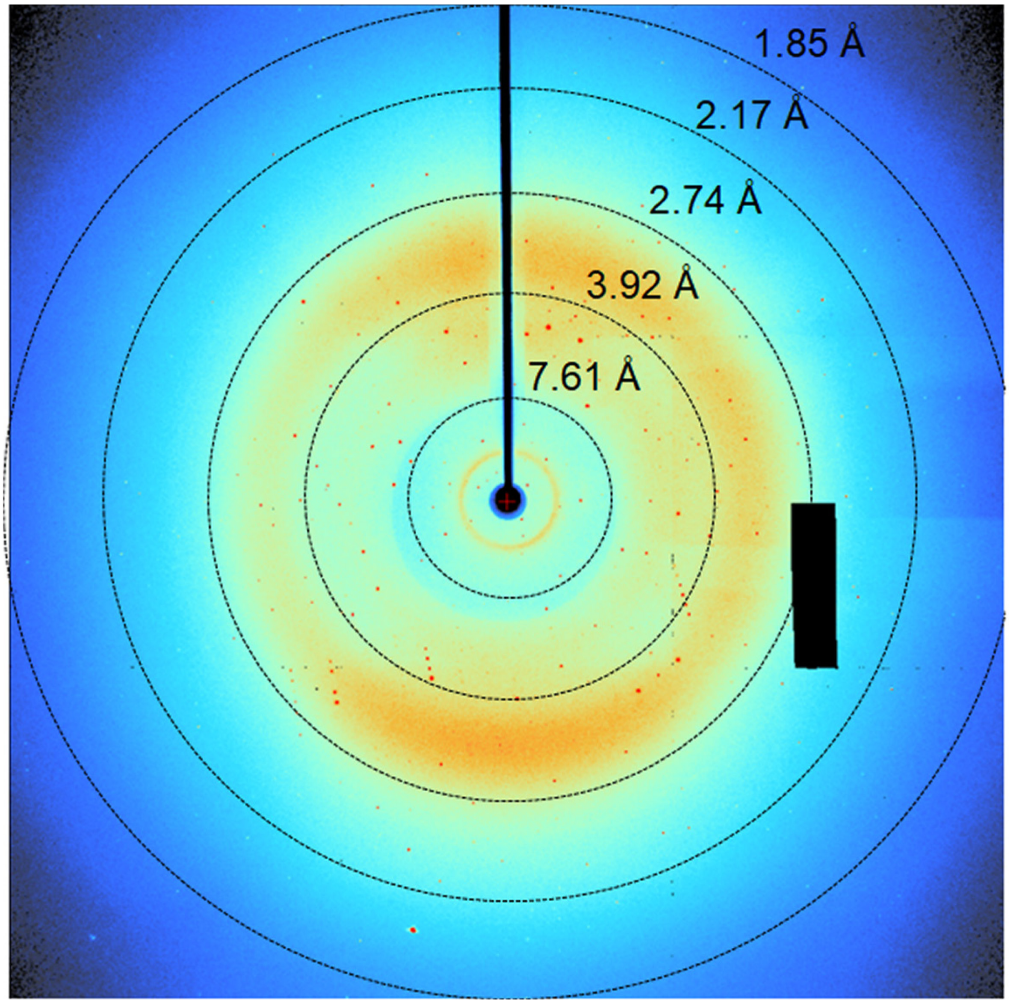

Figure S10 X-ray diffraction quality of the 5-µm lysozyme microcrystals in an environment with high salt condition (3.5 M NaCl). The image shows a relatively low background intensity in the MCC (Fig. 4) compared to that in the LCP injector. The XFEL illumination position is at the centre of the tubing.

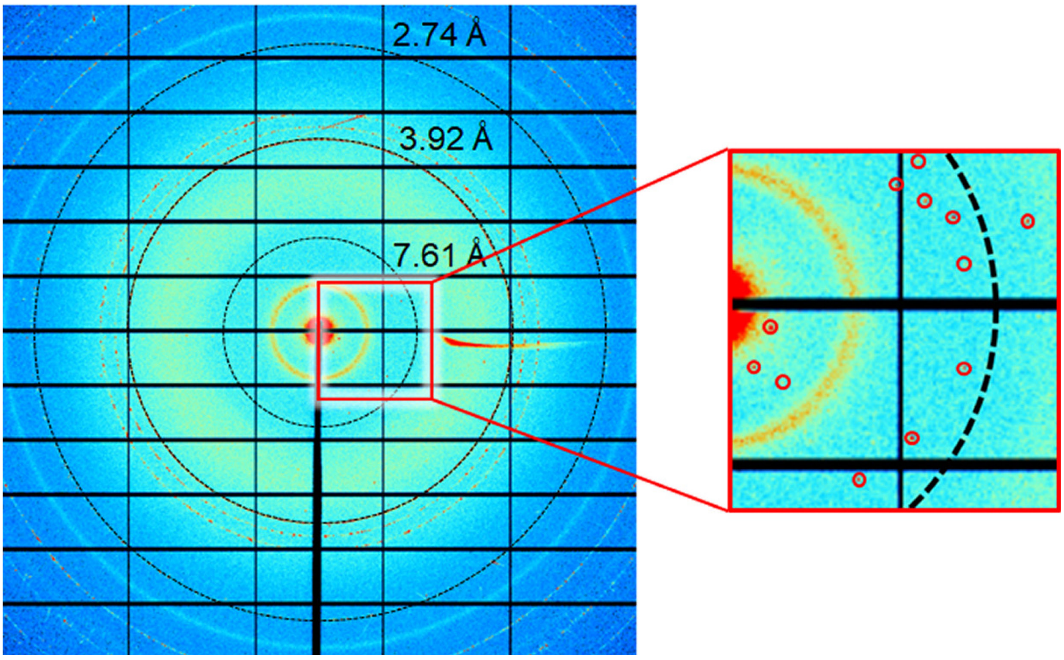

Figure S11 X-ray exposure experiment of proteinase K contained in the MCC with cryocooling (~ 100 K). The diffraction image was collected at the BL-11C beamline [PLS-II (Republic of Korea)] using a Pilatus 6M CCD detector.
